# Linearised and non-linearised isotherm models optimization analysis by error functions and statistical means

**DOI:** 10.1186/2052-336X-12-92

**Published:** 2014-06-10

**Authors:** Busetty Subramanyam, Ashutosh Das

**Affiliations:** 1 School of Civil Engineering, SASTRA University, Thanjavur, Tamil Nadu, India; 2 Director, Centre for Environmental Engineering, PRIST University, Tamil Nadu, India

**Keywords:** Adsorption, Equilibrium, Error function, Statistical function

## Abstract

In adsorption study, to describe sorption process and evaluation of best-fitting isotherm model is a key analysis to investigate the theoretical hypothesis. Hence, numerous statistically analysis have been extensively used to estimate validity of the experimental equilibrium adsorption values with the predicted equilibrium values. Several statistical error analysis were carried out. In the present study, the following statistical analysis were carried out to evaluate the adsorption isotherm model fitness, like the Pearson correlation, the coefficient of determination and the Chi-square test, have been used. The ANOVA test was carried out for evaluating significance of various error functions and also coefficient of dispersion were evaluated for linearised and non-linearised models. The adsorption of phenol onto natural soil (Local name Kalathur soil) was carried out, in batch mode at 30 ± 20 C. For estimating the isotherm parameters, to get a holistic view of the analysis the models were compared between linear and non-linear isotherm models. The result reveled that, among above mentioned error functions and statistical functions were designed to determine the best fitting isotherm.

## Introduction

Phenols are primarily present in oil refinery, coal, paper, textile, synthetic rubber and pharmaceutical wastewaters and are an problem of a serious environmental due to their high toxicity and potential accretion in the environment. There are numerous methods for removing phenols from the wastewaters, such as reverse osmosis, adsorption, bio-degradation, chemical oxidation and solvent extraction. Adsorption is an efficient treatment system for removing phenols from wastewaters.

Adsorption isotherms experimental data is necessary for the design of sorbate-sorbent. To remove phenols from wastewaters a definite sorbate-sorbent system has required to optimize the design and also experimental equilibrium data is important to establish the most appropriate correlation. Kumar and Sivanesan has been studied comparison of linear and non-linear methods for removal of safranin onto rice husk. Subramanyam and Das has been studied comparison of linear and non-linear isotherm models, adsorption of phenol onto natural soil (Kalathur soil). The various adsorption isotherm equation to remove pollutants from wastewaters as have been used to study the nature of adsorption, with the fundamental idea of optimization of the design parameters. Many most commonly used isotherms models appearing in the adsorption literature including, Langmuir, Freundlich and Redlich–Peterson isotherm models [1,2]. All these isotherm models derived based on the theoretical assumption, to measure the goodness of fit in the literature there are number of statistical error deviation functions such as the Marquardt’s percent standard error deviation (MPSED), the correlation coefficient (r^2^), the sum of the squares of the errors (SSE), the hybrid fractional error function (HYBRID), the average relative error deviation (ARED) and the residual analysis (RESID) [3]. However, the very approach of linearization of the nonlinear models necessarily yields rationalization of specific variables, which may have significant bearings on the adsorption process itself.

The main objective of this study was to explore, the applicability of the statistical methods in determining the best fitting isotherm models. The statistical tools used in the present study were Pearson correlation coefficient (r), the coefficient of determination (r^2^), the Chi square test (χ^2^) and ANOVA test. The single component sorption study was carried out based on experiment and computed phenol-sorption by the soil selected (namely, Kalathur soil) on phenol from its aqueous solution to estimate the effect of linearization on the accuracy of the model (on comparison with experimental values).

## Material and methods

The soil (namely Kalathur soil) sample was collected from Thanjavur districts, Tamil Nadu (India). The samples, thus obtained, was washed thoroughly with distilled water and dried for 2 hours, at 105°C in an electric oven, followed by crushing and sieving (100–635 SIEVE NO ASTM E11-87), to obtain the uniform size of particles. The final sample, after passing through the sieve, was dried, desiccated and preserved in air-tight chamber for subsequent analysis and experiments. Soil texture analysis was carried out to find the percentage of sand (25%), silt (20%) and clay (55%) present in the soil. According to United States Department of Agriculture (USDA) texture triangle Kalathur soil (Kr) was classified as clayey soil.

Phenol (C_6_H_5_OH) of analytical reagent (AR) grade supplied by Ranbaxi Laboratories Ltd., India, was used for the preparation of synthetic adsorbate of concentration 100 mg/l. The required quantity of phenol was accurately weighed and dissolved in distilled water and make up to one liter. Fresh stock solution was prepared every day and stored in a brown color glass bottles to prevent photo-oxidation.

To study the equilibrium, batch experiments were conducted at room temperature (30 ± 2°C) for an adsorption period of 24 hours. The effect of adsorbent dosage on the uptake of phenol on to the soil (namely Kalathur soil) was studied at different adsorbent doses (50 to 1000 mg/100 ml) for the concentration of 100 mg/l. The percentage phenol removal and equilibrium adsorption uptake, q_e_ (mg/g), was computed by making use of the equation as given below:

Percent removal =

(1) $$\frac{100\left(C_{o}-C_{e}\right)}{C_{o}}$$

Adsorbed amount (mg/g)

(2) $$q_{e}=\frac{\left(C_{o}-C_{e}\right)V}{w}$$

Where C_0_ is the initial phenol concentration (mg/l), C_e_ the equilibrium phenol concentration (mg/l), V the volume of phenol solution (l) and w is the soil mass of the adsorbent (g).

### Estimation of best-fitting isotherm model

#### Error functions

Average relative error deviation (ARED) is to minimize the distribution in fractional error over the entire range of concentration studied [3].

(3) $$\mathrm{ARED}=\frac{1}{N}\sum \left|\frac{Q_{e,\mathrm{cal}}-Q_{e,\exp}}{Q_{e,\exp}}\right|\;X\;100$$

This error function has a major drawback, inspite of that most of the researchers prefer using this error function. At higher end of the liquid state concentration rage, the calculated isotherm parameters obtained from such error function will yield a better fit. This has been resulted because of the magnitude of the errors and therefore the error function will increase as concentration increases.

(4) $$\mathrm{SSE}=\sum {\left(Q_{e,\mathrm{cal}}-Q_{e,\exp}\right)}^{2}$$

The hybrid fractional error function (HYBRID): To improve the sum of the squares of the errors at lower levels of liquid-phase concentration, this error function was developed. In this task, each the sum of the squares of the error values was divided by the theoretical adsorbent phase concentration value.

(5) $$\mathrm{HYBRID}=\frac{1}{N-P}\sum \left|\frac{Q_{e,\exp}-Q_{e,\mathrm{cal}}}{Q_{e,\exp}}\right|\;X\;100$$

The Marquardt’s percent standard deviation (MPSED): This error function distribution follows the geometric mean error which allows for the number of degrees of freedom of the system.

(6) MPSED=∑Qexp−QcalQexp2N−PX100

The sum of the absolute errors (EABS): It is similar to SSE and provides a better fit at higher concentration for the isotherm parameters.

(7) $$\mathrm{EABS}=\sum_{i=1}^{p}\left|Q_{e,\exp}-Q_{e,\mathrm{cal}}\right|$$

### Statistical functions

Correlation coefficient of Pearson (r): It is a sampling index, shows the degree linearity of between two dependent data series. The degree of linearity varies from −1 to 1.

(8) $$r=\frac{N\left(\sum \mathrm{XY}\right)-\left(\sum X\right)\;\left(\sum Y\right)}{\sqrt{\left[N\;\sum X^{2}-{\left(\sum X\right)}^{2}\right]}\;\left[N\sum Y^{2}-{\left(\sum Y\right)}^{2}\right]}$$

Coefficient of determination (r^2^): It explains the regression line with percentage of variability in the dependent data series variable. The percentage degree varies from 0 to 1.

(9) $$r^{2}=\frac{S^{2}}{S_{\left(\mathrm{XY}\right)}S_{\left(\mathrm{YY}\right)}}$$

Where S_XY_ is the sum of squares of X and Y, S_XX_ is the sum of squares of X and S_YY_ is the sum of squares of Y. In addition to above mentioned error and statistical functions Chi-square test, was also examined to predict best-fitting isotherm models.

The ANOVA test (two factors without replication) was carried out for evaluating significance of various error-functions and four coefficients of dispersion (namely, Coefficient of Range, Coefficient of Quartile Deviation, Coefficient of Mean Deviation, Coefficient of Variation) were evaluated, separately for linearised and non-linearised models. In case of linearised models, only one type of Langmuir distribution (i.e., type-1) was considered because of low mean and lowest variance. A paired t-test was also carried out between the dispersion coefficients of linearised and non-linearised models to evaluate the t-statistics.

## Results and discussion

**Figure 1:**
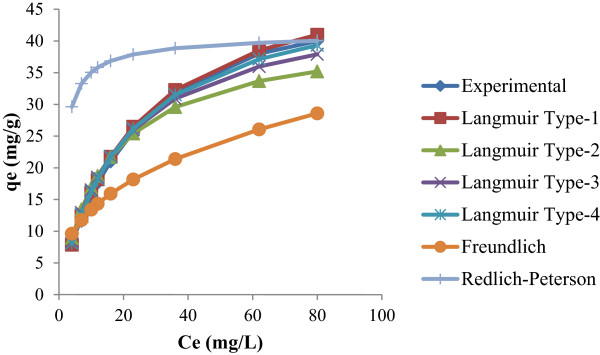
Linearised iostherm models for phenol adsoprtion by Kalathur soil.

**Figure 2:**
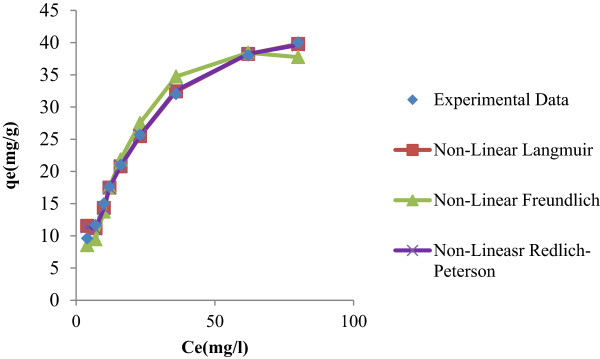
Non-Linearised iostherm models for phenol adsoprtion by Kalathur soil.

In the present study, to find out the isotherm models (linear and non-linearized isotherm) that can describe with precision the experimental results of adsorption isotherms compare the parameters that can be determined (linear and non-linearized isotherm) and also determine the theoretical adsorption isotherms. To remove phenol from liquid phase it is necessary to develop a relationship between a sorption-sorbate system and equilibrium data. Three most commonly used isotherms (viz. Langmuir, Freundlich isotherm and Redlich-Peterson equation) were studied. Table 1 shows isotherm models that are used in the present work and their associated parameters are given Tables 2 and 3 for both linear isotherm and non-linear isotherm analysis. Figure 1 shows the fitting values of linear regression and Figure 2 shows fitting values of non-linear regression analysis.

**Table 1:** Linear and non-linear isotherm equation

**Isotherm**	**Non-Linear equation**	**Linear equation**
Langmuir-1		$\frac{C_{e}}{q_{e}}=\frac{C_{e}}{q_{m}}+\frac{1}{{\mathrm{bq}}_{e}}$
Langmuir-2	$q_{e}=\frac{q_{m\;}{\mathrm{bC}}_{e}}{1+{\mathrm{bC}}_{e}}$	$\frac{1}{q_{e}}=\left(\frac{1}{{\mathrm{bq}}_{m}}\right)\frac{1}{C_{e}}+\frac{1}{q_{m}}$
Langmuir-3		$q_{e}=q_{m}-\left(\frac{1}{b}\right)\frac{q_{e}}{C_{e}}$
Langmuir-4		$\frac{q_{e}}{C_{e}}={\mathrm{bq}}_{m}-{\mathrm{bq}}_{e}$
Freundlich	$q_{e}=K_{F}C_{e}^{1/n}$	$\log\left(q_{e}\right)=\log\left(K_{F}\right)+\frac{1}{n}\log\left(C_{e}\right)$
Redlich-Peterson	$q_{e}=\frac{K_{\mathrm{Rp}\;}C_{e}}{\left[1+\alpha_{\mathrm{RP}}{\left(C_{e}\right)}^{\beta }\right]}$	$\ln\left(\frac{K_{\mathrm{RP}}}{q_{e}}-1\right)=\mathrm{\beta ln}\left(C_{e}\right)+\ln\left(\alpha_{\mathrm{RP}}\right)$

**Table 2:** Linearised isotherm parameters

**Isotherm**	**Kalathur soil**
Langmuir-1	
qm	52.63
b	0.044
Langmuir-2	
qm	41.67
b	0.068
Langmuir-3	
qm	46.52
b	0.055
Langmuir-4	
qm	49.37
b	0.049
Freundlich	
KF	5.801
n	2.747
Redlich-Peterson	
Krp	30.345
β	0.987
α	0.789

**Table 3:** Non-linearised isotherm parameters

**Non-linearized isotherm**	**Kalathur soil**
Langmuir	
qm	51.83
b	0.04333
R2	0.9952
Freundlich	
KF	5.635
n	2.175
R2	0.9953
Redlich-Peterson	
Krp	2.351
β	0.9634
α	0.05369
R2	0.9953

Incorporation of the ANOVA study for the error functions with regard to the isotherm models (for both linear as well as non-linear models) to estimate the significance of variance (refer to Tables 4 and 5). The model compatibility (for both linear and non-linear models) was estimated for the error functions, with regard to not only the lowest value of error, but also the other coefficients of dispersion (refer to Table 4).

**Table 4:** Variation of mean & dispersion coefficients between linearised and Non-linear models

	**Errors-- > statistics**	**ARED**	**MPSED**	**HYBRID**	**SSE**	**RESID**	**EABS**
**Linearised model**							
mean		22.0	27.9	22.0	437.0	10.2	35.4
Dispersion	Coefficient of Range	0.9	0.9	0.9	1.0	2.6	0.9
	Coefficient of Quartile Deviation	0.5	0.5	0.5	0.9	−3.6	0.7
	Coefficient of Mean Deviation	1.0	1.0	1.0	1.3	3.5	0.9
	Coefficient of Variation	1.5	1.5	1.5	1.9	5.5	1.2
**Non-linear model**							
mean		4.9	7.5	4.9	11.2	0.1	7.3
Dispersion	Coefficient of Range	0.3	0.1	0.3	0.7	−8.4	0.5
	Coefficient of Quartile Deviation	0.2	0.1	0.2	0.5	1.8	0.3
	Coefficient of Mean Deviation	0.3	0.1	0.3	0.8	5.9	0.5
	Coefficient of Variation	0.4	0.2	0.4	1.0	7.7	0.6

**Table 5:** ANNOVA

***Source of variation***	***df***	***Sum of squares***		***Mean sum of square***		***F-statistics***		***P-value***	
		***Linearised***	***Non-linear***	***Linearised***	***Non-linear***	***Linearised***	***Non-linear***	***Linearised***	***Non-linear***
Among isotherms	2	600394.12	134.21	300197.06	67.11	1.74	3.73	0.23	0.06
Between error Functions	5	1627971.12	204.89	325594.22	40.98	1.88	2.28	0.18	0.13
Residual Error	10	1727359.62	179.72	172735.96	17.97				

### Error analysis

#### Linear isotherm

The linearized four forms of Langmuir isotherm model were presented in Table 1[4] and the Langmuir coefficients for four linearized Langmuir equation were obtained by plotting graphs between Ce/qe versus Ce (Type- I linearized equation), 1/qe versus 1/Ce (Type- II linearized equation), qe versus qe/Ce (Type- III linearized equation), and qe/Ce versus qe (Type- IV linearized equation). Table 2 shows the calculated parameters of the four linearized Langmuir isotherm model. A graph was drawn between experimental and observed data as shown in Figure 2. From Table 2, it can be inferred that, different linear Langmuir equations show different Langmuir constants, as indicated by variation in errors, specific to the corresponding mode of linearization [5]. In the case of Kalathur soil, on comparison of the four linearized Langmuir equations, it is observed that the Type- I linearized Langmuir equation showed higher value of correlation coefficient (r2 = 0.994) than that of the other three linearized equations (Type- II to IV) as shown in Table 2. The adsorption capacity of Kalathur soil was found to be 52.63 mg/g for Type –I linearized Langmuir and that of Type- II, III and IV are 41.67 mg/g, 46.52 mg/g and 49.37 mg/g, respectively. Thus, during linearization, errors in the computation of parameters may be responsible for the variation in adsorption capacity ‘q_m_’ and adsorption constant ‘b’ (from Type-I linearized isotherm form to Type-IV linearized isotherm). In other words, the transformation of non-linear isotherm model to linear isotherm models seems to implicitly alter the error functions as well as the error variance and normality assumptions of the least-squares methods [4,6,7]. As suggested in the lower correlation coefficient values, it will be inappropriate to use this type of linearization.

In order to verify the validity of the linearized and non-linearised isotherm models as well as the best-fitting isotherm model, six common statistical error methods were employed to calculate the error divergence between observed and predicted sorbate-sorbent system data. It is clear from the Table 6 that the linearized Langmuir models (Type-I, Type-II, Type-III and Type-IV), Freundlich and Redlich-Peterson isotherms, Langmuir isotherm Type-1 shows higher r^2^ value and low error values (i.e., ARED, HYBRID and EABS). Which indicates among all the linearised isotherm form of the Langmuir isotherm type-I is able to describe equilibrium data and indicates the best linearised isotherm model.

**Table 6:** Error functions

**Error function type/isotherm model**	**ARED**	**MPSED**	**HYBRID**	**SSE**	**RESID**	**EABS**
Linear						
Langmuir						
Type-I	**5.156953**	7.148837	**5.156953**	7.578923	4.022454	**7.469256**
Type-II	8.543072	9.621322	8.543072	56.61154	−6.95591	17.98949
Type-III	6.432829	7.416404	6.432829	16.64189	**−1.62588**	11.22502
Type-IV	5.219617	**6.884058**	5.219617	**7.380351**	0.566875	7.610797
Freundlich	19.68531	23.10193	19.68531	481.0593	−51.2595	51.58342
Redlich-Peterson	86.76697	113.0963	86.76697	2053.002	116.6088	116.6088
Non-Linear						
Langmuir	**3.722937**	7.060828	**3.722937**	4.801368	0.716535	**4.474066**
Freundlich	7.189632	8.87458	7.189632	24.15513	**−0.91032**	12.61618
Redlich-Peterson	3.777602	**6.685821**	3.777602	**4.70022**	0.541658	4.739453

The bold letters indicates lowest values of ARED, MPSED, HYBRID, SSE, RESID and EABS.

Redlich-Peterson isotherm (using three-parametric modeling equation: Table 2) was plotted using experimental data between ln(KrpCe/qe −1) and ln(Ce). Redlich-Peterson isotherm contains three unknown variables (viz. Krp, α and β), it is not possible to obtain three unknown variables using linearising Redlich-Peterson isotherm. Thus, the three unknown variables were obtained by minimization of the isotherm equation (and, thus, by maximization of the correlation coefficient) (Tables 6 and 7). In this case, the calculated parameters need not be unique (and could reflect the local optima) and hence the comparison of linearised and non-linear isotherms may not be relevant. In order to verify the model validity the correlation coefficient was lower, as well as the ARED, HYBRID, EABS value are very high (when compare other four linearized Langmuir and Freundlich). Therefore, the Redlich-Peterson linearised isotherm model fails to explain the sorbate-sorbent system of phenol onto Kalathur soil.

**Table 7:** Statistical function

**Statistical function type/isotherm model**	**Pearson correlation coefficient**	**Determination coefficient**	**Chi square**
Linear			
Langmuir			
Type-I	0.9419	**0.994**	**0.388526**
Type-II	0.9149	0.951	0.598918
Type-III	0.9287	0.891	2.003552
Type-IV	0.9357	0.891	0.78796
Freundlich	**0.9759**	0.979	20.96659
Redlich-Peterson	0.7951	0.98	60.37647
Non-Linear			
Langmuir	0.9485	**0.9952**	0.388526
Freundlich	0.8955	**0.9953**	1.246057
Redlich-Peterson	0.9453	**0.9953**	**0.368326**

The bold letters indicates highest values of Pearson Correction coefficient, determination Coefficient as well as lowest Chi Square.

### Non-linear isotherm

In the present study, for studying non-linear isotherm models, Graph Pad Prism versions 5.0 have been used for determining the non-linear coefficients. The determined coefficients were shown in Table 3. Correspondingly, a plot was drawn between Ce versus qe (Figure 2) using the experimental and predicted value by non-linear models. From Tables 6 and 7, it was observed that the correlation coefficient value is high (r ^2^ = 0.9953) and the low ARED, HYBRID and EABS values, thus it indicates that the models are able describe equilibrium data perfectly. Therefore, as far as the non-linear isotherm model is concerned the error remains constant. Hence, to use the correlation coefficient values for comparing the best-fitting non-linear isotherm models is befitting.

Table 3 shows a non-linear Langmuir, Freundlich and Redlich-Peterson model parameter values and Figure 2 shows a plot between Ce versus qe . The correlation coefficient value higher than that of linearized isotherm model. Table 6, shows error function value. But, the error values show the improved up on linearized isotherm.

In the case of Langmuir and Redlich-Peterson model, the model shows high correlation coefficients value. The error functions ARED, HYBRID and EABS was found to be good for Langmuir isotherm model and error functions MPSED and SSE was found to be good for Redlich-Peterson isotherm models. It is clear from above results the Langmuir isotherm and Redlich-Peterson model were for better than Freundlich isotherm model. The models were able describe experimental data perfectly. Hence, it can be understood that, the Redlich–Peterson and Langmuir isotherms were the most suitable models for sorbate-sorbent system. A close correspondence was found to exist between Langmuir and Redlich-Peterson isotherm models. Similar findings have also been reported by other researchers as well [3,4,8,9].

As can be seen from Table 7, best fitting isotherm model was determined more appropriately by the Chi-square test. However, among the three isotherm models studied in this work, the phenol adsorption onto soil system was appropriately explained by Redlich-Peterson isotherm model only. Indeed the transformation of non-linear to linear models misrepresents the experimental error, which limits the validity of the error function and statistical tools. To avoid the errors discussed above, non-linear regression method can be more appropriately used.

As per the ANOVA table, although the p-values for isotherms is higher in case of linearised model than in case of non-linear models, indicating the relatively higher significance level associated with non-linearised case than the linearised case, yet at 0.05 significant level the variation is less than the table values for F-distributions. In fact, the variability between error functions is even less significant. These indicate that the selection of error functions and the isotherm models are fairly unbiased estimators for linearised and nonlinear models (Tables 4 and 5).

The overall mean of the errors of the linearised models is 93.5% higher than that of the non-linear models. To evaluate dispersion from the mean value, four coefficients of dispersion were studied, which indicated that 87.5% time the linearised models show higher dispersion coefficient. In fact, paired t-test results show that the confidence level for the non-linear models (as higher than linearised models) is more than 84%.

## Conclusions

Thus based on the statistical studies it was found that variability in both linearised and non-linear cases are not significant at 0.05 significant level (both among isotherms and among error functions) confirming the error-estimators & isotherm models used as fairly unbiased (yet non-linear models do have relatively higher significance compared to linearised models, though). The overall mean of the error functions of linearised models is significantly higher (and so also most of the dispersion coefficients), compared to their non-linear counterparts, indicating non-linear modeling to be much better representation of experimental results than the linearised ones.

## Competing interest

The authors declare that they have no competing interests.

## Authors’ contributions

SB, carried out all the experimental work and design of the experiments, AD took part in the design of the experiments, supervision of the PhD work. Both the authors read and approved the final manuscript.
